# Human Tissue Kallikrein Activity in Angiographically Documented Chronic Stable Coronary Artery Disease

**DOI:** 10.5935/abc.20150109

**Published:** 2015-11

**Authors:** Estêvão Lanna Figueiredo, Carolina Antunes Magalhães, Karlyse Claudino Belli, Ari Mandil, José Carlos Faria Garcia, Rosanã Aparecida Araújo, Amintas Fabiano de Souza Figueiredo, Lucia Campos Pellanda

**Affiliations:** 1 Departamento de Cardiologia – Hospital Lifecenter, Belo Horizonte, MG; 2 Departamento de Análises Clínicas e Toxicológicas – Faculdade de Farmácia – Universidade Federal de Minas Gerais (UFMG), Belo Horizonte, MG; 3 Laboratório de Pesquisa de Patofisiologia do Exercício – Divisão de Cardiologia – Hospital de Clínicas de Porto Alegre, Porto Alegre, RS; 4 Research on Research Network Team – Duke University, North Carolina, USA; 5 Departamento de Hemodinâmica – Hospital Lifecenter, Belo Horizonte, MG; 6 Programa de Pós-Graduação em Cardiologia – Fundação Universitária de Cardiologia (PPGFUC/RS), Porto Alegre, RS; 7 Universidade Federal de Ciências da Saúde de Porto Alegre (UFCSPA), Porto Alegre, RS – Brazil

**Keywords:** Human Tissue Kallikrein, Tissue Kallikrein, Kallikrein-Kinin System, Coronary Artery Disease

## Abstract

**Background:**

Human tissue kallikrein (hK1) is a key enzyme in the kallikrein–kinin system (KKS). hK1-specific amidase activity is reduced in urine samples from hypertensive and heart failure (HF) patients. The pathophysiologic role of hK1 in coronary artery disease (CAD) remains unclear.

**Objective:**

To evaluate hK1-specific amidase activity in the urine of CAD patients

**Methods:**

Sixty-five individuals (18–75 years) who underwent cardiac catheterism (CATH) were included. Random midstream urine samples were collected immediately before CATH. Patients were classified in two groups according to the presence of coronary lesions: CAD (43 patients) and non-CAD (22 patients). hK1 amidase activity was estimated using the chromogenic substrate D-Val-Leu-Arg-Nan. Creatinine was determined using Jaffé’s method. Urinary hK1-specific amidase activity was expressed as *µ*M/(min · mg creatinine) to correct for differences in urine flow rates.

**Results:**

Urinary hK1-specific amidase activity levels were similar between CAD [0.146 *µ*M/(min ·mg creatinine)] and non-CAD [0.189 *µ*M/(min . mg creatinine)] patients (p = 0.803) and remained similar to values previously reported for hypertensive patients [0.210 *µ*M/(min . mg creatinine)] and HF patients [0.104 *µ*M/(min . mg creatinine)]. CAD severity and hypertension were not observed to significantly affect urinary hK1-specific amidase activity.

**Conclusion:**

CAD patients had low levels of urinary hK1-specific amidase activity, suggesting that renal KKS activity may be reduced in patients with this disease.

## Introduction

Coronary artery disease (CAD) caused approximately 1 out of every 6 deaths in the United States in 2007 ^1^. An estimated 16,300,000 Americans have CAD, of which one of the most prevalent risk factors (RFs) is hypertension^1^. Blood pressure exhibits an inverse relationship with urinary or renal human tissue kallikrein (hK1) activity levels in primary hypertensive patients^2-4^. In addition, urinary hK1 levels are significantly reduced in heart failure (HF) patients ^5-7^.

Kallikreins (EC 3.4.21.8) comprise a subgroup of the serine protease family and are known to have several physiological functions, including the control of blood pressure, coronary artery perfusion, electrolyte balance, inflammation, and other diverse physiological processes^2,7-9^. Our group has demonstrated that urinary hK1-specific amidase activity is significantly reduced in hypertension and HF patients^3,6^.

Hypertension is a RF for both CAD and HF, and HF is the final outcome of untreated hypertension and CAD. However, urinary hK1 levels and their association with the severity of angiographically determined stable CAD remains unknown. The aim of the present study was to evaluate hK1-specific amidase activity in the urine of established or suspected CAD patients.

## Methods

We conducted a cross-sectional study from January 2008 to January 2010. Sixty-five individuals were enrolled in the study regardless of gender and race. Random midstream urine samples were collected in the Catheterism Laboratory at the Lifecenter Hospital immediately before cardiac catheterism (CATH) for known or suspected CAD.

This study was approved by the Ethical Research Committees of both the Lifecenter Hospital in Belo Horizonte and the Federal University of Minas Gerais, as well as the National Commission for Ethics in Research. All patients read the protocol, had their queries satisfactorily replied, and provided written informed consent.

Urine samples were transferred to the Clinical Enzymology Laboratory of the Clinical Chemistry Department, Pharmacy School, Federal University of Minas Gerais, and were visually and chemically examined using a dipstick test (Urofita 10U bioBRÁS Diagnósticos, Biobrás S.A., Belo Horizonte, MG, Brazil). All urine samples were negative for all evaluated chemical compounds (e.g., bilirubin, blood, glucose, ketone bodies, nitrites, protein, urobilinogen).

Traditional RFs for CAD were identified through standard interviews conducted directly by physicians, as well as by nurse and pharmacist participating in the study. CATH exams were conducted by interventional cardiologists certified by the Brazilian Society of Interventional Cardiology, and were registered in the hospital records. Patients underwent a thorough clinical interview and physical examination. All symptoms and signs were analyzed, as well as patients’ personal histories and the use and types of cardiovascular or non-cardiovascular medications. All subjects were studied as outpatients.

### Inclusion and exclusion criteria

Patients of any gender and race were eligible for inclusion in the study if they were older than 18 years and younger than 75 years, had known or suspected CAD, and provided consent. Suspected CAD was defined by the medical history, physical examination, electrocardiogram, abnormalities in other imaging exams (e.g., exercise test, echocardiogram, and myocardial scintigraphy). The criteria for patient exclusion were non-agreement for study participation, recent history of acute coronary syndrome (< 3 months), serum creatinine level ≥ 1.5 mg/dL or 133 *µ*mol/L for men and ≥ 1.4 mg/dL or 124 *µ*mol/L for women, history of severe allergy to ionic contrast agents, or presence of blood or nitrite in the urine.

### Presence and severity of CAD

Patients were classified according to the presence or absence of CAD. Diagnostic coronary angiograms were performed under local anesthesia (90% femoral approach and 10% radial) with non-ionic contrast agent and were classified by either of the two examiners through a visual analysis of stenosis as mild (< 40%), moderate (40-70%), and severe (70-100%); flow was classified according to the Thrombolysis In Myocardial Infarction Group criteria^10^. Questionable cases were reviewed by both examiners, sometimes with the aid of quantitative coronary angiography (Axiom Artis, Siemens, Munich, Germany), and/or were referred for intravascular ultrasonography (IVUS; I-LAB Boston Scientific, Natick, MA, USA). Both examiners were blinded to the hK1-specific amidase activity results.

### hK1 amidase activity

hK1 amidase activity was evaluated using the chromogenic substrate D-Val-Leu-Arg-Nan (Chromogenix AB, Italy)^11^. Substrate hydrolysis was assayed spectrophotometrically at 410 nm to monitor the release of 4-nitroaniline (4-Nan) [ε_410_ = 8800/(M · cm)], as previously described^12^. The assay was performed as previously described^6^. Specifically, 5 incubation mixtures, identified by capital letters A, B, C, D, and E, contained the following: A, 500 *µ*L of urine and 100 *µ*L of 200 mM glycine-NaOH buffer, pH 9.0 containing 0.05% (w/v) NaN_3_ (Sigma Chemical Co., St. Louis, MO, USA); B, 500 *µ*L of urine and 100 *µ*L of a 1000 KIU/mL Trasylol^®^ solution (strong kallikrein inhibitor also known as aprotinin, bovine pancreatic trypsin inhibitor-BPTI, or Kunitz pancreatic trypsin inhibitor)^11^; C, 500 *µ*L of urine and 100 *µ*L of a 1 mg/mL SBTI (Sigma) solution (strong serine proteinase and plasma kallikrein inhibitor that is not, however, an hK1 inhibitor)^6^ in 200 mM of glycine-NaOH buffer, pH 9.0; D, 500 *µ*L of urine and 100 *µ*L of 200 mM glycine-NaOH buffer, pH 9.0; and E, 600 *µ*L of 200 mM glycine-NaOH buffer, pH 9.0. The mixtures were preincubated at 37ºC for 10 minutes for temperature equilibration. Next, 400 *µ*L of a 160 *µ*M D-Val-Leu-Arg-Nan substrate solution in 200 mM glycine-NaOH buffer, pH 9.0, were added to the A, B, C, and E mixtures; 400 *µ*L of 200 mM glycine-NaOH buffer, pH 9.0, was added to the D mixture instead of substrate solution. The mixtures were again incubated at 37ºC for 30 minutes, and the reactions were stopped by the addition of 100 *µ*L of 60% (v/v) acetic acid per mixture. The mixtures were incubated in quadruplicate. Enzymatic hydrolysis was monitored by measuring the absorbance at 410 nm of the released 4-Nan using a Shimadzu UV 160 A UV-Vis recording spectrophotometer (2-nm spectral band width). The reference cell contained 1000 *µ*L of glycine-NaOH buffer, pH 9.0, and 100 *µ*L of acetic acid solution. The total substrate concentration was determined from the amount of 4-Nan released after complete hydrolysis by an excess of bovine β-trypsin (kindly provided by Dr. Marcelo Matos Santoro, Departamento de Bioquímica e Imunologia, ICB, UFMG, Belo Horizonte, MG, Brazil). The ∆A_410_ values were calculated and transformed into reaction rates (v) and expressed as *µ*M/(min · mL urine). The reaction rates were linear up to 60 minutes.

In all evaluated urine samples, the enzymatic activity was completely inhibited by Trasylol^®^ (incubation mixture B); no inhibition was observed in the presence of SBTI (incubation mixture C), indicating only the presence of the enzyme hK1.

hK1-specific amidase activity was calculated by dividing the reaction rate (v) by the creatinine concentration (mg/mL urine). The result was expressed as *µ*M/(min · mg creatinine) to correct for differences in the urine flow rate^13^.

### Creatinine determination

Creatinine was determined spectrophotometrically using a kit of reagents based on Jaffe’s reaction (Bioclin/Quibasa Química Básica Ltda, Belo Horizonte, MG, Brazil) and expressed as mg/mL urine, as described in our previous reports^3,6,13^. These assays were also performed in quadruplicate.

### Confounders

Information about patients’ medication use was obtained through interviews. Drugs were classified into 2 groups: angiotensin converting enzyme inhibitors (ACEI)/angiotensin receptor blockers (ARB) and all other medications. Blood pressure was measured in all patients while at rest and in a sitting position prior to the CATH procedure; this information was classified in the interview according to the international guidelines^14-16^.

### Statistical analysis

Data were analyzed using Minitab software for Windows, version 15.0, and are expressed as medians because of the irregular distributions of the investigated variables. Differences between groups and the effects of medications on hK1-specific amidase activity in CAD were evaluated using the non-parametric Mann-Whitney test, as the studied population exhibited a non-Gaussian distribution with non-homogeneous variances. Differences between CAD severity subgroups were compared using the non-parametric Kruskal-Wallis test. The frequencies of both genders and the presence or absence of hypertension, angina pectoris, dyspnea, stroke, and diabetes mellitus were compared using the Chi-square test. The presence or absence of gout and hypothyroidism were compared using Fisher’s test. A p**value < 0.05 was considered statistically significant.

The sample size was calculated based on values determined during our previous studies [difference between groups: HF patients, 0.104 *µ*M/(min · mg creatinine) and controls, 0.260 *µ*M/(min · mg creatinine)] with a standard deviation of 0.23; an estimated 35 patients were needed for each group at a significance level of 0.95 and power of 0.8.

This study was developed within the Research and Innovation Coaching Program, a partnership between the Brazilian Society of Cardiology and the Duke University Research on Research group (USA)^17^.

## Results

**Figure 1:**
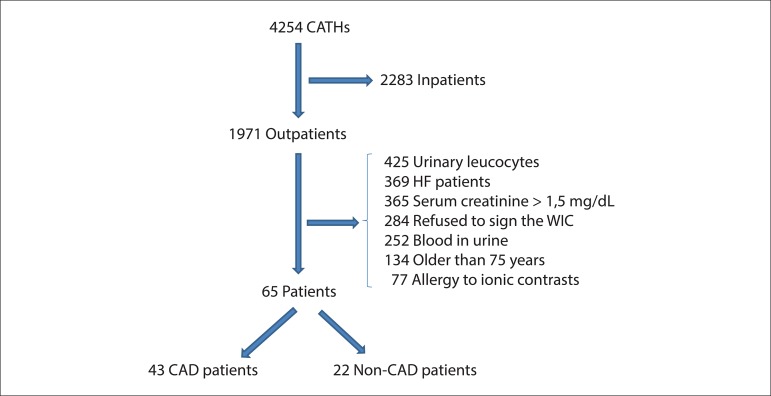
Flowchart of eligible and included patients in the present study. CATH: Cardiac catheterism; HF: Heart failure; WIC: Written informed consent; CAD: Coronary artery disease.

From January 2008 to January 2010, 4254 CATHs were performed at Lifecenter Hospital; of these, 65 treated patients were included in this study (Figure 1). Patients were classified into 2 groups according to the presence of coronary lesions: CAD (43 patients), and non-CAD (22 patients). The subgroups were similar with respect to gender and the presence or absence of hypertension. Although CAD patients were significantly older than non-CAD patients, patients with and without hypertension did not differ in terms of age. Among the 43 CAD patients, 36 were hypertensive (Table 1). Among the 22 non-CAD patients, 19 were hypertensive (Table 1).

**Table 1:** Baseline characteristics of the studied patients

**Parameter**	**CAD Patients (n = 43)**	**Non-CAD Patients (n = 22)**	**P**
**Demographic**			
Age (years)^a^	62.5 (55.0-69.0)	56.7 (49.3-64.3)	0.029^b^
Gender (male/female)	30/13	10/12	0.057^c^
**Physiologic**			
Hypertensive (yes/no)	36/07	19/03	0.085^c^
Angina pectoris (yes/no)	18/25	2/20	0.007^c^
Dyspnea (yes/no)	4/39	4/18	0.516^c^
Stroke (yes/no)	0/43	1/21	0.338^c^
Diabetes mellitus (yes/no)	6/37	1/21	0.478^c^
Gout (yes/no)	1/42	0/22	1.000^d^
Hypothyroidism (yes/no)	1/42	0/22	1.000^d^
**Biochemical**			
hK1 sp am act a,e	0.146 (0.085-0.260)	0.189 (0.069-0.323)	0.803^b^

CAD: Coronary artery disease; Non-CAD: Non coronary artery disease; hK1 sp am act: hK1-specific amidase activity;

a Median values; numbers in parentheses are the 25–75% interquartile ranges;

b Mann–Whitney test;

c Chi-Square test;

d Fisher’s test;

e μM/(min · mg creatinine).

A p value < 0.05 was considered statistically significant.

Among the 43 CAD patients, the median hK1-specific amidase activity was 0.146 *µ*M/(min · mg creatinine), whereas among the 22 non-CAD subjects this value was 0.189 *µ*M/(min · mg creatinine).

CAD and non-CAD patients did not differ significantly with respect to urinary hK1-specific amidase activity (Table 1).

Among the 43 CAD patients, 14, 6, and 23 had mild, moderate, and severe stenosis, respectively (Table 2). We did not observe any statistically significant effect of CAD severity on urinary hK1-specific amidase activity (Table 2).

**Table 2:** Urinary hK1-specific amidase activity and CAD classification

**Parameter**	**Mild (n = 14)**	**Moderate (n = 06)**	**Severe (n = 23)**	**p**
hK1 sp am act a^,^^b^	0.181 (0.097-0.413)	0.245 (0.119-0.545)	0.141 (0.069-0.264)	0.234^c^

CAD: Coronary artery disease; hK1 sp am act: hK1-specific amidase activity;

a μM/(min . mg creatinine);

b Median values; numbers in parentheses are the 25-75% interquartile ranges;

c Kruskal-Wallis test.

A p value < 0.05 was considered statistically significant

No effect of hypertension on urinary hK1-specific amidase activity was observed in either CAD or non-CAD patients (Table 3).

**Table 3:** Effect of hypertension on hK1-specific amidase activity in CAD and Non-CAD patients

	**CAD (n=43)**			**Non-CAD (n=22)**		
	**Hyp (n=26)**	**Non-Hyp (n=17)**	**p**	**Hyp (n=10)**	**Non-Hyp (n=12)**	**p**
hK1 sp am act a^,^^b^	0.139	0.245	0.785^c^	0.202	0.148	0.531^c^
	(0.091-0.250)	(0.066-0.323)		(0.064-0.537)	(0.070-0.266)	

CAD: Coronary artery disease; Non-CAD: Non-coronary artery disease; Hyp: Hypertensive; Non-Hyp: Non-hypertensive; hK1-sp am act: hK1 specific amidase activity;

a μM/(min. mg creatinine);

b Median values: numbers in prenthesis are the 25-75% interquartile ranges;

c Mann-Whitney test. A p value < 0.05 was considered statistically significant

Regarding medication use, 16 CAD patients were using ACEI/ARB drugs. There was no statistically significant difference in ACEI or ARB use with respect to urinary hK1-specific amidase activity (Table 4).

**Table 4:** Effects of medications on hK1-specific amidase activity in CAD patients

	**ACEI/ARB (n = 16)**	**Other medications (n = 27)**	**p**
hK1 sp am act a^,^^b^	0.179 (0.089-0.259)	0.153 (0.069-0.291)	0.900^c^

ACEI/ARB: Angiotensin converting enzyme inhibitor/angiotensin II receptorblocker; hK1 sp am activity: hK1-specific amidase activity;

a μM/(min . mg creatinine);

b Median value; numbers in parentheses are the 25-75% interquartile ranges;

c Mann-Whitney test. A p value < 0.05 was considered statistically significant.

The urinary hK1 specific amidase activity values in CAD patients were within the ranges described previously for HF and hypertensive patients^3,6^ (Table 5).

**Table 5:** Median hKI-specific amidase activity values In heart failure, hypertensive, CAD, and Non-CAD patients

**Parameter**	**HF Patients^a^ (n = 28)**	**CAD Patients (n = 43)**	**Non-CAD Patients (n = 22)**	**Hyp Patients^b^ (n = 100)**	**HF Controls^a^ (n = 28)**	**Hyp Controls^b^ (n = 89)**
hK1 sp am act c^,^^d^	0.104 (0.067-0.297)	0.146 (0.085-0.280)	0.189 (0.069-0.323)	0.210 (0.100-0.395)	0.213 (0.147-0.401)	0.260 (0.180-0.445)

HF: Heart failure; CAD: Coronary artery disease; Non-CAD: Non-coronary artery disease; Hyp: Hypertensive; hK1 sp am act: hK1-specific amidase activity;

a Median values described in Figueiredo et al^6^;

b Median values described in Belo et al^3^;

c μM/(min • mg creatinine;

d Median value; numbers in parentheses are the 25-75% interquartile ranges.

## Discussion

To the best of our knowledge, this is the first study to compare urinary hK1-specific amidase activity in human patients with and without angiographically documented stable CAD.

We evaluated hK1-specific amidase activity in the urine of CAD and non-CAD patients and found no statistically significant difference between the groups.

Kallikreins (EC 3.4.21.8) comprise a subgroup of the serine protease family and known to have several physiological functions. These proteins are divided into 2 main groups: plasma (EC 3.4.21.34) and tissue (EC 3.4.21.35) kallikreins^2^. The human KLK1 gene, located on chromosome 19q13.4, expresses true tissue kallikrein (hK1) in the kidney, pancreas, salivary glands, vasculature, and heart, among other tissues^2,8,9,11^. The best known biochemical function of hK1 is release of the vasoactive and spasmogenic decapeptide kallidin (lysyl-bradykinin) (Lys-BK) from the plasma protein low molecular weight kininogen; Lys-BK is involved in the control of blood pressure, coronary artery perfusion, electrolyte balance, inflammation, and other diverse physiological processes^2,7-9^. hK1 activity can be measured in the urine using photometric assays with synthetic substrates, or radioimmunoassays^11^. Many studies concerning the role of KKS in heart and circulatory diseases also address kinins^2,18,19^.

In 2006, we reported the results of a study of 28 HF patients and 28 healthy control subjects in whom the median urinary hK1 specific amidase activity was significantly lower among HF patients [0.104 *µ*M/(min · mg creatinine)] than among controls [0.213 *µ*M/(min · mg creatinine)] (p = 0.020)^6^.

In 2009, our group also reported a study of 100 non-diabetic primary hypertensive patients and 89 healthy control subjects, in which the median value of urinary hK1-specific amidase activity was significantly lower in hypertensive patients [0.210 *µ*M/(min · mg creatinine)] than in controls [0.260 *µ*M/(min . mg creatinine)] (p = 0.010)^3^.

In the present study, urinary hK1-specific amidase activity did not significantly differ between CAD and non-CAD patients (Table 1). For CAD patients, the median value of urinary hK1-specific amidase activity observed in the present study [0.146 *µ*M/(min · mg creatinine)] remained within the values previously reported for HF [0.104 *µ*M/(min · mg creatinine)] and hypertensive [0.210 *µ*M/(min · mg creatinine)] patients of our previous studies^6,3^.

On the other hand, the median value of urinary hK1-specific amidase activity for non-CAD subjects in the present study [0.189 *µ*M/(min · mg creatinine)] was lower than the median values reported for the controls in our previous studies of HF patients [0.213 *µ*M/min · mg creatinine] and hypertensive patients [0.260 *µ*M/(min · mg creatinine)]^6,3^ (Table 5).

For further analysis, we compared the median values of urinary hK1-specific amidase activities of HF [0.104 *µ*M/(min · mg creatinine)] and hypertensive patients [0.210 *µ*M/(min · mg creatinine)] of our previous studies^6,3^ with the value reported herein for CAD patients [0.146 *µ*M/(min · mg creatinine)]. We observed no significant differences in these comparisons (p = 0.297 and p = 0.131, respectively). We also compared the median urinary hK1 amidase activities for HF [0.104 *µ*M/(min · mg creatinine)] and hypertensive patients [0.210 *µ*M/(min · mg creatinine)] in our previous studies^6,3^ with the value reported herein for non-CAD patients [0.189 *µ*M/(min · mg creatinine)]. Again, no significant differences were observed (p = 0.629 and p = 0.184, respectively). Unlike our previous studies, wherein the controls were asymptomatic and lacked known diseases^3,6^, in the present study, all subjects who underwent CATH exhibited symptoms, signs, or suspected CAD. We know that we cannot exclude endothelial or microvascular disease in angiographically normal coronary arteries, and that normal or near-normal coronary angiograms are observed in up to 20% of women with documented myocardial ischemia^20-22^. Therefore, we suggest that this type of disease might explain the lack of differences in urinary hK1-specific amidase activities between the 2 groups of patients (CAD and non-CAD).

The results reported in the present study demonstrate****that the median value of urinary hK1-specific amidase activity of CAD patients remained within the values previously described for hypertensive and HF patients^3,6^ (Table 5). As hK1-specific amidase activity was reduced among patients in those studies, relative to normal controls, we suggest that hK1-specific amidase activity might also be reduced in the group of CAD patients studied herein. We must also consider the fact that our non-CAD group underwent CATH. Therefore, although the non-CAD group lacked angiographically documented CAD, they differed from a completely healthy, disease-free population.

Nolly et al^23^ reported the presence of a local KKS in rat hearts and suggested that locally generated kinins might help regulate cardiac function.

Some studies have produced direct evidence for a cardioprotective role of tissue kallikrein in infarcted rats.

In 2002, Agata et al^24^ used a somatic approach to explore the role of the KKS in cardiac remodeling and apoptosis after myocardial infarction (MI) in rats. Rats were submitted to coronary artery ligation to induce MI, and adenovirus carrying the hK1 or luciferase (control) gene was injected into the tail vein at 1 week after surgery. Cardiac output gradually decreased from 2 to 6 weeks after MI, whereas delivery of the kallikrein gene prevented this decrease.

In 2005, Griol-Charhbili et al^25^ tested the hypothesis that tissue kallikrein (TK) would play a protective role in myocardial ischemia by inducing ischemia-reperfusion (IR) injuries with and without ischemic preconditioning (IPC) or ACE inhibitor (ramiprilat) pretreatment *in vivo* in littermate wild-type (WT) or TK-deficient (TK^-/-^) mice. IR induced similar infarcts in WT and TK^-/-^ mice. IPC reduced the infarct size by 65% in WT mice and by 40% in TK^-/-^ mice (P < 0.05, TK^-/-^ *vs.* WT). Although ramiprilat also reduced the infarct size by 29% in WT, its effect was completely suppressed in TK^-/-^ mice. Pretreatment of WT mice with a B2, but not a B1, kinin receptor antagonist reproduced the effects of TK deficiency. However, B2 receptor-deficient mice (B2^-/-^) unexpectedly responded to IPC or ramiprilat similarly to WT mice. However, constitutively high levels of B1 receptor gene expression were observed in B2^-/-^ mice following pretreatment. In WT and TK^-/-^ mice, both the B2 and B1 mRNA levels increased several fold during IR and increased further still during IPC + IR. Thus, according to the authors, TK and the B2 receptor play critical roles in cardioprotection against ischemia following 2 experimental and potentially clinically relevant maneuvers.

In 2006, Koch et al^26^ reported a study in which the bradykinin coronary outflow, left ventricular performance, and left ventricular dimensions were investigated in transgenic rats harboring the hK1 gene (hKLK1) under basal and ischemic conditions. The main finding of their study was that transgenic rats harboring hKLK1, which were characterized by increased basal coronary bradykinin levels, demonstrated improved cardiac function and remodeling after MI induction in vivo.

In the same year, Spillmann et al^27^ induced MI in anesthetized mice by permanently occluding the left anterior descendant coronary artery. hKLK1 was delivered to the peri-infarct myocardium via an adenoviral vector (Ad.hKLK1). Controls received empty vector (Ad.Null) or saline. The survival rates were similar among the groups. Ad.hKLK1 increased the number of circulating endothelial progenitor cells and promoted the growth of capillaries and arterioles in the peri-infarct myocardium. In addition, Ad.hKLK1 increased the number of cardiac progenitor cells in the peri-infarct and suppressed the apoptotic death of peri-infarct cardiomyocytes both in vivo and ex vivo. As a consequence of these beneficial effects, hKLK1-transduced hearts were protected from post-MI ventricular dilatation and showed better systolic and diastolic functions at 5 weeks after MI. Similar results were reported by Pons et al^28^ in 2008.

In 2007, Yao et al^29^ reported a study in which they examined the potential therapeutic effects of a consistent, subdepressive dose of infused kallikrein and kinin on ventricular remodeling and neovascularization in rats after MI. At 1 week after coronary artery ligation, tissue kallikrein (TK) or bradykinin (BK) was infused through a minipump for 4 weeks. At 5 weeks after MI, this TK or BK infusion significantly improved cardiac contractility and reduced diastolic dysfunction without affecting systolic blood pressure. The infusions also significantly increased capillary density in the non-infarcted region. The TK and BK infusions also reduced the heart weight/body weight ratio, cardiomyocyte size, and atrial natriuretic peptide and brain natriuretic peptide expression levels in the non-infarcted area. The authors concluded that a subdepressive dose of kallikrein or BK could restore impaired cardiac function in rats with post-infarction HF by inhibiting hypertrophy and fibrosis and promoting angiogenesis through increased nitric oxide formation and suppression of oxidative stress and TGF-β1 expression.

In 2008, Chao et al^30^ investigated the role of TK in protection against cardiac injury mediated through direct kinin B2 receptor activation in kininogen-deficient Brown Norway Katholiek rats after inducing acute MI. TK was injected locally into the myocardium of Brown Norway Katholiek rats after coronary artery ligation with and without coinjection of icatibant (a kinin B2 receptor antagonist) and N^ω^-nitro-L-arginine methyl ester (a nitric oxide synthase inhibitor). One day after MI, TK treatment significantly improved cardiac contractility and reduced the MI size and left ventricle and diastolic pressure in Brown Norway Katholiek rats. Kallikrein attenuated ischemia-induced apoptosis and monocyte/macrophage accumulation in the ischemic myocardium, in conjunction with increased nitric oxide levels and reduced myeloperoxidase activity. Icatibant and N^ω^-nitro-L-arginine methyl ester abolished the effects of kallikrein, indicating a kinin-B2 receptor-nitric oxide-mediated event. All of these studies demonstrated the beneficial effects of KKS in animals with acute MI (a severe form of CAD), but none measured tissue kallikrein activities in infarcted animals, as we did in stable CAD and non-CAD human patients. As treatment with injected TK or BK improved cardiac function and prevented HF in ischemic conditions in rats in previous studies, we could suppose that tissue kallikrein activity was also reduced in those animals.

In 2013, Yao et al^31^ evaluated whether the levels of both tissue kallikrein and the inflammatory biomarker high-sensitivity C-reactive protein (hs-CRP) in the peripheral blood would correlate with plaque stability, as well as the relationship among tissue kallikrein expression, macrophage numbers, and angiogenesis in CAD. Tissue kallikrein, vascular endothelial growth factor (VEGF), and hs-CRP plasma levels were measured in 100 patients newly diagnosed with CAD and 33 CAD-free controls. CAD patients were defined as having angiographically diagnosed coronary stenosis amounting to a lumen reduction of at least 50% or greater coronary diameter lumen stenosis in a major coronary artery. CAD patients were further divided according to the number of diseased vessels into single-vessel CAD, multi-vessel CAD, and multi-vessel CAD with acute obstruction of 1 major coronary artery groups. hK1 plasma levels were determined using an ELISA specific for hK1. Patients were stratified according to CAD severity as moderate (n = 33), multi-vessel (n = 35), and multi-vessel with acute coronary syndromes (ACS; n = 32). Patients without CAD were used as controls (n = 33). The authors found that patients with CAD and ACS had significantly elevated levels of tissue kallikrein. In addition, the hs-CRP concentration was increased in patients with ACS. A strong positive correlation between plasma tissue kallilkrein and CAD severity was also demonstrated. The incidence of major adverse cardiovascular events (MACE) during an 8- to 24-month follow-up period significantly correlated with tissue kallikrein levels in the ACS group. The authors concluded that plasma TK levels were a useful predictor for the presence and extent of CAD.

In 2014, Zhang et al^32^ investigated the relationship between plasma tissue kallikrein levels and the presence and severity of CAD in a Chinese population. The study involved 898 consecutive CAD patients and 905 ethnically and geographically matched controls. CAD was angiographically confirmed in all patients, and the severity of CAD was determined according to the number of affected vessels and coronary stenosis scores. Plasma tissue kallikrein levels, which were measured via ELISA, were significantly higher in CAD patients than in controls (0.347 ± 0.082 vs. 0.256 ± 0.087 mg/L, p < 0.001) and were directly associated with a higher risk of CAD (odds ratio = 3.49, 95% confidence interval: 2.90-4.19). The authors themselves affirmed that paradoxically, the plasma tissue kalllikrein level was independently and positively associated with the presence of human CAD, although numerous studies (as described above) have confirmed the independent cardioprotective effect of tissue kallikrein in animal models. On the other hand, Zhang et al^32^ used a 1-way ANOVA and multivariable stepwise linear regression analysis to demonstrate that plasma tissue kallikrein levels were negatively associated with the severity of CAD, according to vessel scores (p < 0.001) and stenosis scores (r = -0.211, p < 0.001).

In contrast to these 2 earlier studies, we measured the activity of urinary tissue kallikrein, whose origin is renal, using a spectrophotometric method. The former authors measured tissue kallikrein levels, rather than activity, directly in plasma (via ELISA). However, many tissues (e.g., brain, pancreas, salivary glands, heart) are known to produce plasma tissue kallikrein^2^.

We also evaluated the effect of CAD severity on urinary hK1-specific amidase activity. As mentioned above, no effect was observed (Table 2). In our published study regarding urinary hK1-specific amidase activity and HF^6^, we also observed no relationship between HF severity and urinary hK1-specific amidase activity. Yao et al^31^ observed a strong positive correlation between plasma tissue kallilkrein and CAD severity of CAD, whereas Zhang et al^32^ demonstrated the opposite result. Therefore, additional studies are truly needed to understand the true role of hK1 in CAD.

We also evaluated the effect of hypertension on urinary hK1-specific amidase activity in CAD and non-CAD patients to exclude possible confounding factors, and observed no effect (Table 3). Regarding medication use among CAD patients, 16 and 27 patients did and did not use angiotensin-converting-enzyme inhibitors/angiotensin receptor blockers (ACEI/ARB), respectively. We found no statistically significant influence of ACEI or ARB use on urinary hK1-specific amidase activity (Table 4).

The results reported in the present study demonstrate that the urinary hK1-specific amidase activity levels in CAD patients remained within the previously described ranges for HF and hypertensive patients^6,3^ (Table 5). As hK1 specific amidase activity in those studies was reduced in comparison to that of normal controls, we suggest that urinary hK1-specific amidase activity and, consequently, renal KKS activity, might also be reduced in the studied group of CAD patients. We must consider that patients in our non-CAD group also underwent CATH. Therefore, although the patients in this group were angiographically diagnosed as free of CAD, they might differ from a completely healthy, disease-free population.

### Study limitations

This study has some limitations. First, it is cross-sectional and therefore cannot be used to infer causality; however, our aim was to test our hypothesis regarding an association between hK1 and CAD. Second, as observed in ,Figure 1, we evaluated 1971 potentially eligible patients, but could only include 65 (43 with CAD and 22 without CAD) in the study. Despite this fact, the characteristics of our sample were similar to those reported in previous studies of CAD patients^1^. According to our study aim, we needed to include angiographically documented non-CAD patients to provide a comparison of urinary hK1-specific amidase activity levels with those of angiographically documented CAD patients.

## Conclusion

In conclusion, compared with non-CAD controls, the urinary hK1-specific amidase activity was reduced in our population of angiographically documented CAD patients, similar to previously reported findings in HF and hypertensive patients. Although this finding might have been expected, it has not previously been demonstrated. A reduction in urinary hK1-specific amidase activity in CAD patients suggests that renal KKS activity might also be reduced in these patients.
